# Ultrasound-Induced Release of Nimodipine from Drug-Loaded Block Copolymer Micelles: In Vivo Analysis

**DOI:** 10.1007/s12975-021-00979-1

**Published:** 2022-01-05

**Authors:** Katja Döring, Swetlana Sperling, Milena Ninkovic, Henning Schroeder, André Fischer, Christine Stadelmann, Frank Streit, Lutz Binder, Dorothee Mielke, Veit Rohde, Vesna Malinova

**Affiliations:** 1 Department of Neurosurgery, University Medical Center Göttingen, Göttingen, Germany; 2 Department of Neuroradiology, University Medical Center Göttingen, Göttingen, Germany; 3 Department for Epigenetics and System Medicine in Neurodegenerative Diseases, German Center for Neurodegenerative Diseases, Göttingen, Germany; 4 Department of Neuropathology, University Medical Center Göttingen, Göttingen, Germany; 5 Institute for Clinical Chemistry, University Medical Center Göttingen, Göttingen, Germany

**Keywords:** Drug-loaded nanocarrier, Nimodipine, Ultrasound, Cerebral vasospasm treatment

## Abstract

Nimodipine prevents cerebral vasospasm and improves functional outcome after aneurysmal subarachnoid hemorrhage (aSAH). The beneficial effect is limited by low oral bioavailability of nimodipine, which resulted in an increasing use of nanocarriers with sustained intrathecal drug release in order to overcome this limitation. However, this approach facilitates only a continuous and not an on-demand nimodipine release during the peak time of vasospasm development. In this study, we aimed to assess the concept of controlled drug release from nimodipine-loaded copolymers by ultrasound application in the chicken chorioallantoic membrane (CAM) model. Nimodipine-loaded copolymers were produced with the direct dissolution method. Vasospasm of the CAM vessels was induced by means of ultrasound (Physiomed, continuous wave, 3 MHz, 1.0 W/cm^2^). The ultrasound-mediated nimodipine release (Physiomed, continuous wave, 1 MHz, 1.7 W/cm^2^) and its effect on the CAM vessels were evaluated. Measurements of vessel diameter before and after ultrasound-induced nimodipine release were performed using ImageJ. The CAM model could be successfully carried out in all 25 eggs. After vasospasm induction and before drug release, the mean vessel diameter was at 57% (range 44–61%) compared to the baseline diameter (set at 100%). After ultrasound-induced drug release, the mean vessel diameter of spastic vessels increased again to 89% (range 83–91%) of their baseline diameter, which was significant (*p* = 0.0002). We were able to provide a proof of concept for in vivo vasospasm induction by ultrasound application in the CAM model and subsequent resolution by ultrasound-mediated nimodipine release from nanocarriers. This concept merits further evaluation in a rat SAH model.

**Graphical abstract:**

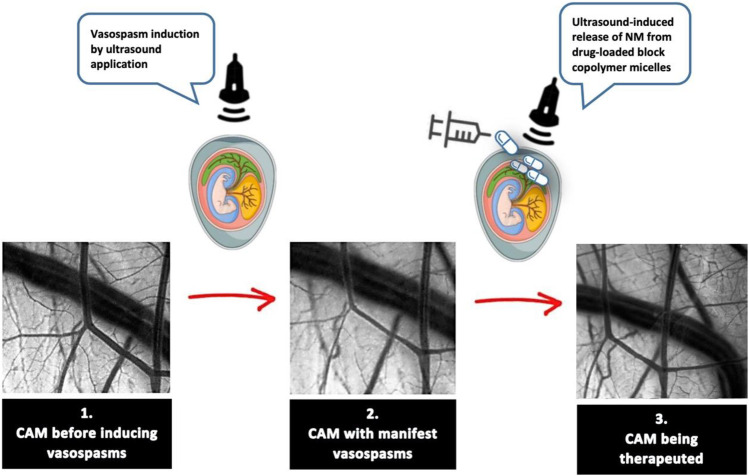

## Introduction

Aneurysmal subarachnoid hemorrhage (aSAH) is a devastating cerebrovascular disease often resulting in poor outcome with permanent disability. Delayed cerebral infarction is one of the main contributors to permanent neurological deficits. Despite continuously growing knowledge concerning the multifactorial pathogenesis of secondary ischemic complications after aSAH, nimodipine remains the only drug with existing evidence not only for vasospasm prevention but also for improvement of functional outcome [1–5]. A prerequisite for this effect is a sufficient intrathecal nimodipine concentration, which is limited by low oral bioavailability of nimodipine [6]. Nanocarrier systems with continuous nimodipine delivery to the cerebrospinal fluid (CSF) have been increasingly evaluated in order to overcome the limitations of systemic nimodipine administration [7, 8]. A sustained intrathecal nimodipine release over 21 days starting with the day of nanocarrier administration has been previously demonstrated [9–12]. Based on existing evidence, higher nimodipine concentrations are deemed necessary during the time peak of vasospasm development, which is between days 5 and 7 after aneurysm rupture [3]. A technique allowing an on-demand drug release from nanocarriers has not been established yet. Pluronic® F-127 block copolymers are built of a hydrophobic core (nano-reservoir) surrounded by polyethylene oxide chains, which is an ideal drug nanocarrier for hydrophobic substances such as nimodipine [13]. The encapsulation of nimodipine into the nano-reservoir rather relies on a physical reaction than on a chemical bond consequently opening the possibility of drug release by external stimuli such as ultrasound application [14]. In this preclinical study, we aimed at evaluating the concept of ultrasound-induced drug release from nimodipine-loaded block copolymers and to demonstrate the drug effect on the chicken chorioallantoic membrane (CAM) vessels in vivo in the absence as well as in the presence of vasospasm.

## Methods

The in vivo experiments involved three core steps: (1) assessment of the nimodipine effect on the CAM vessels in a control group without vasospasm, (2) assessment of the nimodipine-loaded copolymers’ effect on the CAM vessels in the absence of vasospasm, (3) assessment of the nimodipine-loaded copolymers’ effects on CAM vessels with and without drug release in the presence of vasospasm.

### Preparation of Nimodipine-Loaded Pluronic® F-127 Block Copolymers

As nanocarriers, commercially purchased Pluronic® F-127 block copolymers (BASF Corporation, NJ, USA) were used, without additional purification. Nimodipine powder was purchased from Sigma-Aldrich Chemical Company (MO, USA). Nimodipine-loaded block copolymers were produced using the direct dissolution method as previously described [15]. For this purpose, a defined amount of Pluronic® F-127 block copolymer (three different concentrations 5%, 10%, and 15%) and 2 mg nimodipine powder were added simultaneously to 10 mL deionized water and subsequently stirred at 100 rpm and room temperature 25 °C for 3 h. Afterwards, the precipitated nimodipine was separated from the micelle suspension by filtering through 1.0-µm filter membranes (pluriStrainer®, pluriSelect Life Science, Leipzig, Germany).

Particle size measurements, morphological examination, and assessment of the micelles’ stability were conducted by performing an in vitro study for preparation and characterization of nimodipine-loaded Pluronic® F-127 block copolymers [16]. The particle size measurements were performed using a transmission electron microscope (TEM) and nanoparticle tracking analysis (NTA, NanoSight® LM10-HS; Malvern Instruments, Worcestershire, UK; DZNE Göttingen, Germany). The average particle size was less than < 150 nm, which was increasing from 127 to 149 nm. The particle morphology was evaluated under TEM, where the nimodipine-loaded micelles presented with a spherical form and a smooth surface.

The nimodipine-loaded block copolymers show stability over time concerning their size and morphology, after the solutions were stored for 3 months at room temperature and at 4 °C with evaluation of size and morphology by TEM and NTA at defined time points (1 week, 1 month, and 3 months), respectively [16].

### Preparation of the CAM Model

White fertilized eggs of the Bresse Gauloise chicken breed (Poulets-de-France, Hildesheim, Germany) were used. A fortnightly incubation was ensured by the incubator BSS 420, species number 8301/01 (Grumbach® Automatic System GmbH & Co.KG, Nürnberg, Germany). On day 0, the eggs were incubated at 37.7 °C with a constant humidity of 65% according to a previously described technique by Leighton et al. [17]. On day 4 of incubation, the shell was penetrated at the lower pole, in the area of the air chamber. The window was fixed and stabilized by applying Leukotape® (BSN medical, Hamburg, Germany), and then impregnated with a fine needle and fenestrated with scissors. Subsequently, the egg was closed with transparent film (Parafilm® Pechiney Plastic Packaging, OH, USA) until the next step. On day 14, the tape was removed, and the experiments were started after ensuring the survival of the chicken embryo.

### Vasospasm Induction by Ultrasound Application

**Fig. 1:**
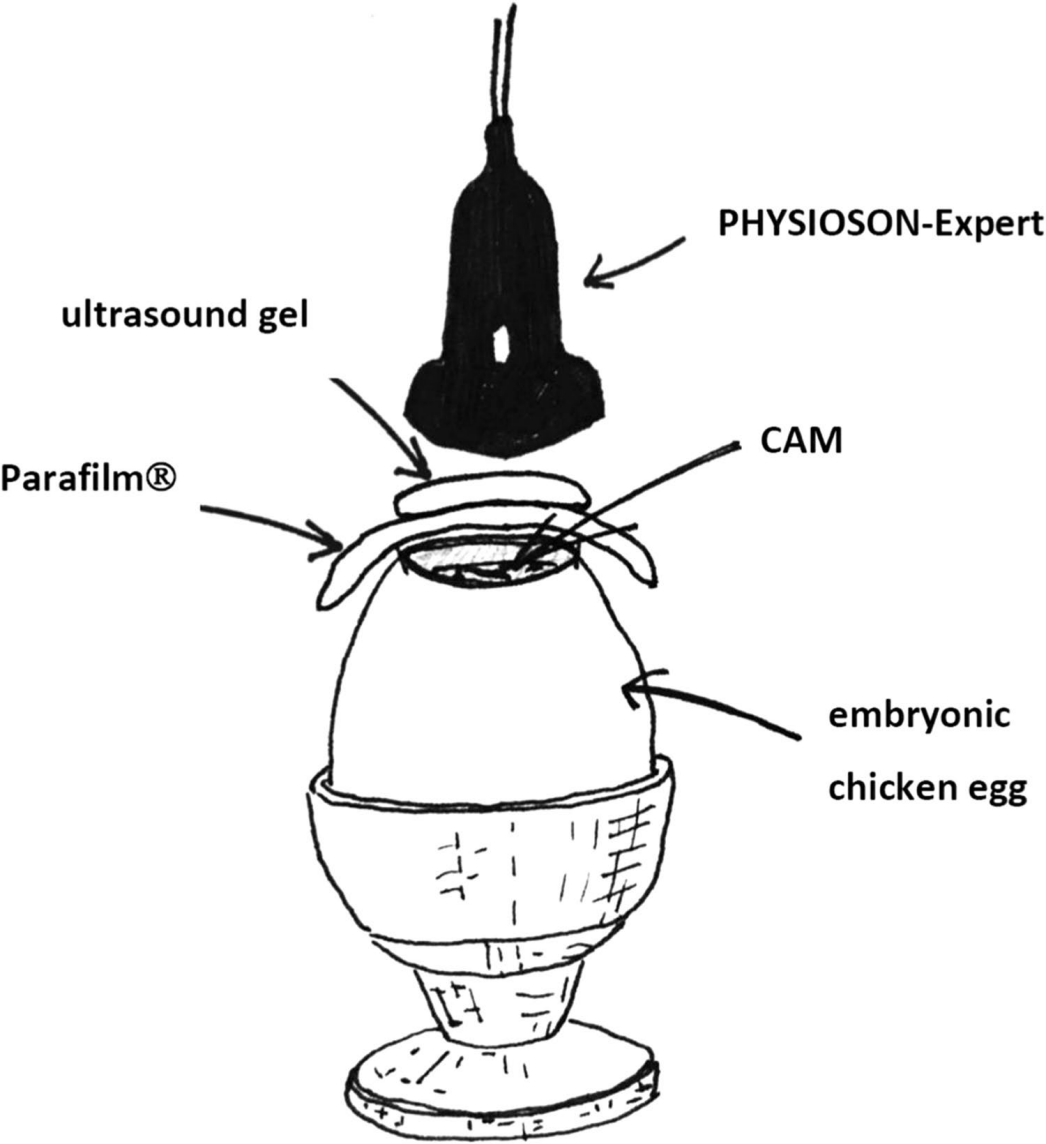
Experimental setup during the ultrasound application to the CAM vessels with the exposed CAM through the egg-windowing covered with Parafilm® and ultrasound gel

The in vivo vasospasm induction in the CAM model is based on the application of ultrasound (continuous wave mode, maximum effective area of 5 cm^2^, 3 MHz frequency, and an intensity of 1.0 W/cm) to the CAM vessels for 60 s. First, the fertilized, incubated eggs were stabilized in a cup. Thermostable ultrasound gel (Sonosid® Asid Bonz GmbH, Herrenberg, Germany) was applied to the CAM protected by Parafilm®. The ultrasound probe was applied directly to the gel (Fig. 1).

### Ultrasound-Controlled Drug Release from the Nimodipine-Loaded Pluronic® F-127 Block Copolymers

The nimodipine-loaded micelles were delivered directly to the CAM exposed through the egg-windowing by using an Eppendorf pipette (1 µL each). The ultrasound treatments were performed with a PHYSIOSON-Expert device (Physiomed ®, Paderborn, Germany). The ultrasound-controlled drug release was done with the same device used for vasospasm induction, but by applying different device settings: continuous wave mode, maximum effective area of 5 cm^2^, 1 MHz frequency, and an intensity of 1.7 W/cm^2^. The ultrasound treatment was applied with a duration of 60 s.

### *Qualitative and Quantitative *In Vivo* Vasospasm Assessment*

The visualization of vessel diameter changes was performed using a 5-MP HD-microscope camera (Leica® MC170 HD, Leica Camera AG, Wetzlar, Germany). The vessel diameter was determined as a quantitative measure at predefined time points using Fiji ImageJ [18]. The values were expressed as arbitrary units according to existing standards [19]. The distance of the ultrasound probe to the vessels remained unchanged in this experimental setting. During the ultrasound application, no microscopic images and monitoring of changes could be made due to the experimental setup. Accordingly, images were taken at 1-min intervals over a period of 20 min and the macroscopic changes were recorded. The measurements were taken on one vessel, reproducibly at the same location each time. The criteria used for qualitative microscopic evaluation were retraction of surrounding CAM and vessel bursting. Additionally, macroscopic examinations were performed by observing the embryonic behavior (alteration in embryonic motility).

### Proof of Effectiveness of the Released Nimodipine from the Nanocarrier

#### Evaluation in a Control Collective

As a control medium, we investigated the effect of nimodipine Carinopharm 0.2 mg/mL, and diluted nimodipine Carinopharm 4 µg/mL (1:50 in deionized water) and pure deionized water on the CAM of three fertilized eggs. For this purpose, 1 µL of each solution was applied directly to the CAM and the effect on the vessels was recorded in 1-min intervals over 20 min using life imaging via a HD-microscope camera.

#### Evaluation in a Collective Without Vasospasm

In the absence of vasospasm, we assessed the effect of nimodipine-loaded copolymers under the following conditions: (1) without ultrasound-guided release, and (2) with ultrasound-guided release of nimodipine from the nanocarrier. The experiments were performed using solutions with varying concentrations (5%, 10%, and 15%). Three embryonic eggs per condition were tested, whereby 1 µL of each solution was applied directly to the CAM. For condition 1, the image was recorded via a microscope camera at 1-min intervals over a period of 20 min. For condition 2, we first applied 1 µL of the solutions, and then took an image and subsequently performed the ultrasound-guided release of the drug using ultrasound (1 MHz, 1.7 W/cm^2^) for 60 s. This was followed by image acquisition at 1-min intervals over a period of 20 min.

#### Evaluation in a Collective With Vasospasm

The evaluation in the presence of vasospasm was performed under the following conditions: (1) without ultrasound-guided release and (2) with ultrasound-guided drug release. Three embryonic eggs were assessed for each condition using 5% and 10% solutions of nimodipine-loaded block copolymers. The 15% solution had a too viscous and gel-like consistency in previous in vitro analyses of our working group. It is, therefore, considered inappropriate for intrathecal application in future experiments, and we refrained from using the 15% solution in this protocol. As soon as the maximum vasospasm was visible, usually around the eighth minute after vasospasm induction, 1 µL of the nimodipine-loaded copolymers was applied, followed by ultrasonic-controlled nimodipine release. This was followed by observation of the nimodipine effect at 1-min intervals until 20 min after induced nimodipine release.

### Statistical Analysis

Statistical analysis was performed using the statistical software GraphPad Prism (Version 9.0, GraphPad Software, San Diego, CA, USA). A *p* value of < 0.05 was considered significant. All data were reported as mean ± SD. An ANOVA was performed for comparisons between the different treatment groups.

## Results

The CAM model could be successfully carried out in all 25 eggs. Vasospasm could be reliably induced in the group with vasospasm including 10 eggs. The collective without vasospasm consisted of 12 eggs. Three eggs were used as control collective.

### Evaluation in a Control Collective

**Fig. 2:**
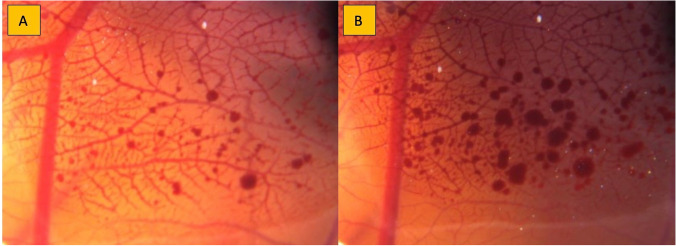
Microscopic recording of the CAM vessels 4 and 10 min after the application of undiluted nimodipine, respectively, depicting the bursting of the vessels **A** 4 min and **B** 10 min after application of undiluted nimodipine S® 0.2 mg/mL

Neither the addition of 1 µL deionized aqua nor the lower concentration (4 µg/mL) of nimodipine Carinopharm led to any microscopically visible and quantitatively measurable alterations in the vessel diameter. In contrast, after application of 1 µL nimodipine Carinopharm 0.2 mg/mL, an almost immediate bursting of the vessels with hemorrhage was observed (Fig. 2).

### Evaluation in a Collective Without Vasospasm

**Fig. 3:**
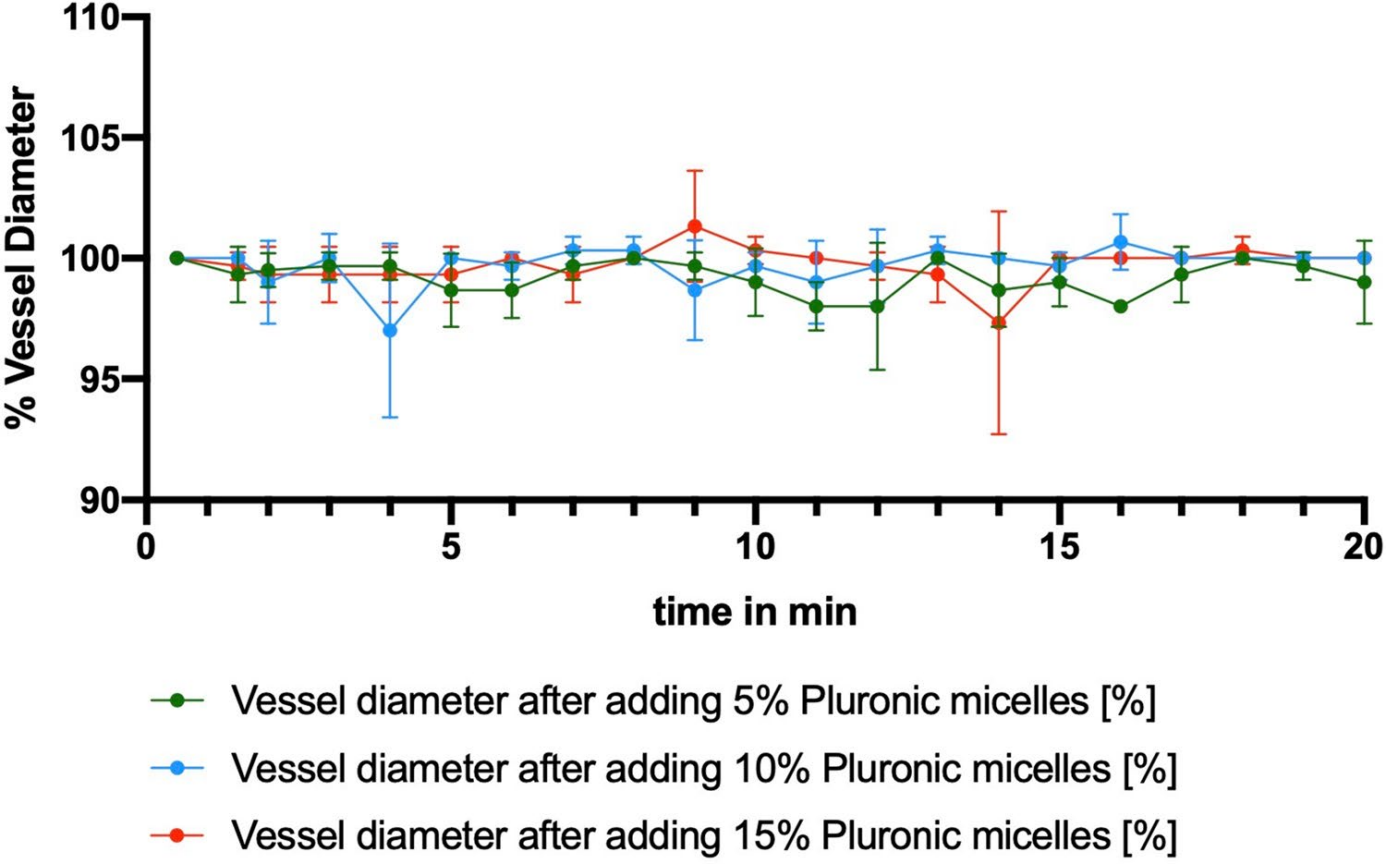
Vessel diameter over the observed period of 20 min after applying the nimodipine-loaded block copolymers with three different concentrations (5%, 10%, and 15%) to a CAM collective without vasospasm, including three eggs per condition (*n* = 3)

Neither a macroscopic change nor a measurable alteration in vessel diameter over the observation period of 20 min was detected when applying the nimodipine-loaded block copolymers to a CAM collective without vasospasm. This was the case in all evaluated solutions (5%, 10%, 15%) of the nimodipine-loaded block copolymers (Fig. 3). The ultrasound-induced drug release also did not lead to any quantifiable or microscopically evaluable changes in vessel diameter.

### Evaluation in a Collective With Vasospasm

**Fig. 4:**
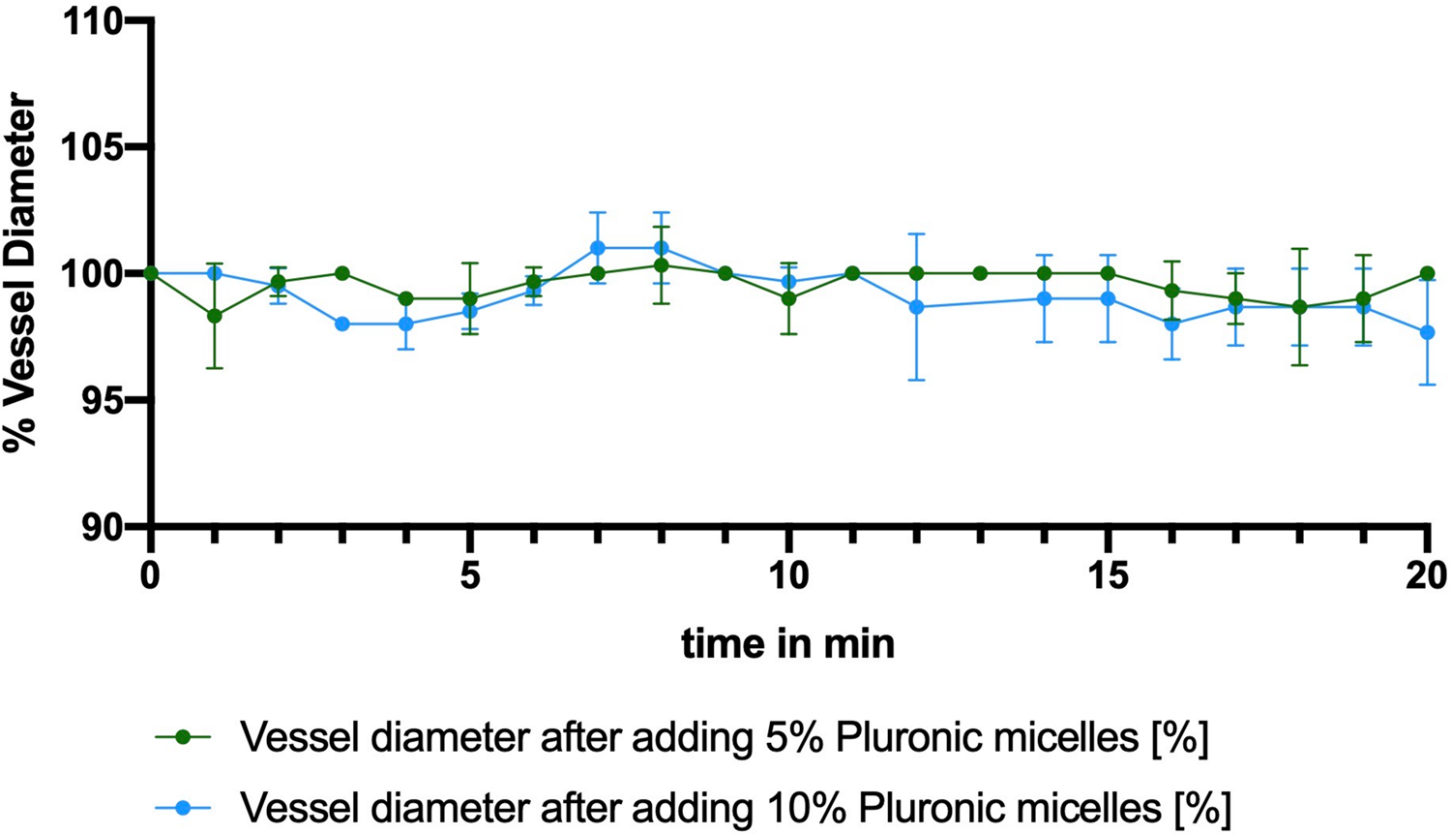
Vessel diameter over the observed period of 20 min after applying the nimodipine-loaded block copolymers with two different concentrations (5% and 10%) to a CAM collective with vasospasm but without drug release, including three eggs per condition

**Fig. 5:**
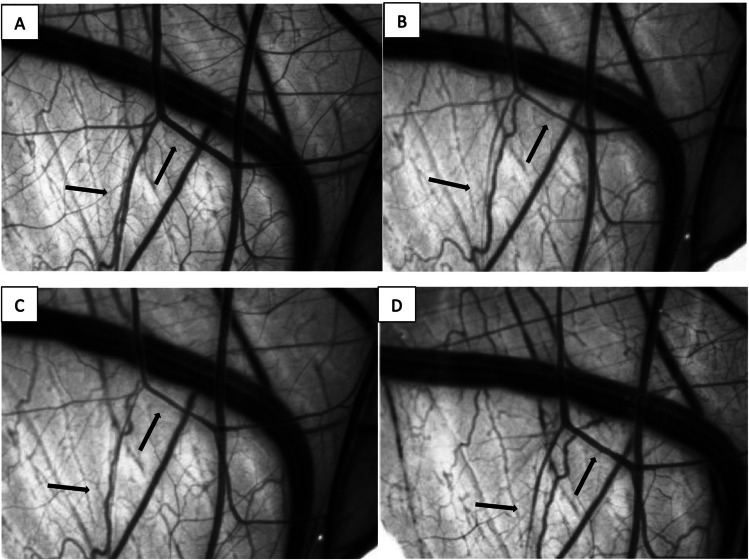
Microscopic recording of the CAM vessels demonstrating the drug effect on VSP after its release from the nanodrug with and without sonification. **A** Depiction of the initial CAM vessels before ultrasound-induced vasospasm. **B** Depiction of the CAM vessels with beginning vasospasm 4 min after sonification. **C** Depiction of the CAM vessels with maximal vasospasm 8 min after sonification. **D** Depiction of the CAM vessels with vasospasm resolution 8 min after ultrasound-mediated nimodipine release

**Fig. 6:**
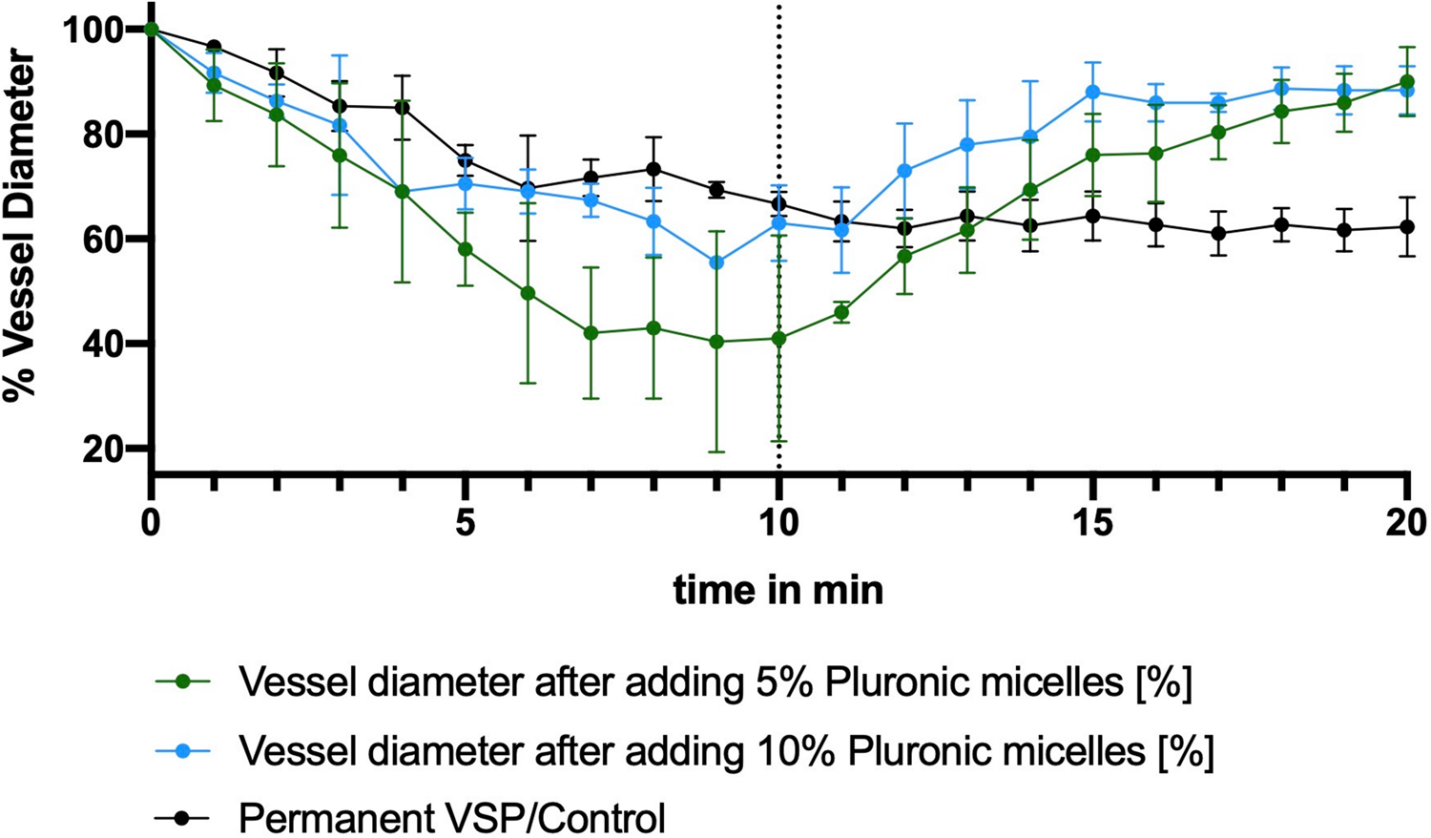
Vessel diameter over the observed period of 20 min after applying the nimodipine-loaded block copolymers with two different concentrations (5% and 10%) to a CAM collective with vasospasm and with ultrasound-induced drug release, including three eggs per condition, compared with a group of three eggs with vasospasm without application of the nimodipine-loaded block copolymers (control group)

After vasospasm induction the mean vessel diameter changed to 57% (ranging from 44 to 61%) compared to the initial vessel diameter, which was set at 100%. Neither microscopically visible nor measurable alterations of the vasospastic vessels could be detected after application of the nimodipine-loaded block copolymer without drug release (Fig. 4). After ultrasound-induced drug release, the mean vessel diameter of vasospastic vessels increased to 89% (range 83–91%) of their baseline diameter, which was statistically significant (ANOVA, *p* = 0.0002). Images of the CAM vessels before and after vasospasm induction as well as before and after ultrasound-induced drug release are demonstrated in Fig. 5. The overall vasospasm resolution rate in the 5% solution was comparable to the vasospasm resolution rate in the 10% solution, when taking into consideration the higher vasospasm severity grade in the 5% solution group compared to the 10% solution group (Fig. 6).

## Discussion

The purpose of this in vivo experimental study was a proof of principle for ultrasound-mediated controlled drug release bound to a nanocarrier. Drug delivery to the central nervous system for treatment of neurological disorders is still challenging due to a limited number of drugs able to pass the blood–brain barrier in a sufficient concentration necessary to deploy a full effect [20, 21]. Intrathecal drug delivery should overcome this limitation of systemically administered drugs. However, intrathecal drug injection has also limitations due to poor drug solubility or pharmacokinetics issues [21]. Especially, hydrophilic substances are cleared quickly after intrathecal injection due to CSF turnover needing repeated dosing to maintain target concentrations. Drug carriers such as nanoparticles may offer a solution with continuous drug release over a long period of time. In the setting of aneurysmal subarachnoid hemorrhage (aSAH), nimodipine is one of the most frequently used drugs for encapsulation into nanoparticles and intrathecal administration with sustained release over time [10, 11]. Considering the course of vasospasm development, however, higher nimodipine concentrations are deemed necessary during the peak time of vasospasm, which is usually between days 5 and 7 after aneurysm rupture [3]. No technique for an on-demand intrathecal nimodipine release from the nanocarrier during the vasospasm peak phase has been established so far. In this study, we showed that nimodipine could be successfully released from nimodipine-loaded block copolymers by applying ultrasound and resolved vasospasm in the in vivo CAM assay. We used, at first, ultrasound to induce vasospasm in the in vivo CAM model following subsequent vasospasm resolution by ultrasound-induced release of nimodipine. To the best of our knowledge, this is the first study demonstrating the concept of ultrasound-mediated vasospasm induction with subsequent ultrasound-induced drug release from nimodipine-loaded Pluronic® F-127 block copolymers consequently resolving vasospasm.

### *The CAM Model Favors the Transition from *In Vitro* to *In Vivo* Studies*

A comprehensive analysis of undesirable effects of nanomaterials and determination of the toxicological profile is necessary before the application of the investigated approach in humans. In this study, we decided to use the CAM model for several reasons: (1) cost-effectiveness; (2) good vascularization; (3) easy to perform as well as repeatable, and (4) direct visualization of vessel changes. The CAM model also most closely resembles the complex conditions of a living organism [22, 23]. According to the 3R principle (reduce, refine, replace), the CAM model seems to be a suitable model for in vivo analysis before the initiation of animal studies [24]. The CAM model offers an uncomplicated setup that requires neither special surgical skills nor a complex surgical environment. After in vivo opening, the eggs do not require any further care as this is the case when using animal models. The low workload of incubating the eggs enables large numbers of test units. In particular, this experimental model allows optimization, standardization, and thus improvement of reproducibility of the in vivo experiments. Furthermore, the free access to the CAM allows a variety of different analyses such as optical evaluation by microscopy as well as further imaging and therapeutical techniques [25]. Here, it enabled us to perform a highly reproducible and comparable qualitative and quantitative evaluation of the CAM vessels. The absence of nociceptive nerves in the CAM and in the chicken embryo up to the 14th day makes the CAM model an ideal alternative to equivalent rodent models. The CAM model thus offers the best conditions to act as a mediator between in vitro and in vivo experiments.

### Ultrasound-Controlled Drug Release from Pluronic® F-127 Block Copolymers

Nanomaterials unveil a variety of potential applications, including their use as drug carriers [26]. As previously described, Pluronic® F-127 copolymers were here used as nanocarrier, which are widely available and considered to be non-toxic [15, 27]. Focused ultrasound emerges as a promising candidate for the active induction of drug delivery systems due to, e.g., its deep soft tissue penetration and its ability to focus its energy on the targeted location of desired drug release [28–30]. Ultrasound can be used to trigger the release of encapsulated drug from copolymers. Although the mechanisms underlying ultrasound-induced drug release from block copolymers are not clear and have to be elucidated in future studies, there is some evidence that mechanical action of ultrasound is involved in this process [28]. Nevertheless, except for our study, no reports currently exist on the use of the CAM model in combination with ultrasound for in vivo vasospasm assessment. We were able to reproducibly induce vasospasm with a diameter reduction of approximately 50% by a short ultrasound application. Due to the overall small CAM vessels, we were not able to induce measurable diameter reduction of more than 50%, which we consider a limitation of the model. The findings of our study are, however, encouraging for further evaluation of the concept in animal models and can be seen as a solid basis for the planning of future experiments.

### Limitations of the Study

There are several limitations of the study that need to be acknowledged here. A general limitation of the CAM model is its short study period. The observation period is limited either by hatching of the embryos at day 21 or by termination of the setup at day 14 due to nociception. Furthermore, the allantoic sac gradually shrinks and loses its function after 19 days of egg hatching with a limited length of research window [31]. Another limitation is that chickens are not mammals. Ultimately, this leads to the consequence that the obtained results cannot be transferred to humans without restrictions [32]. The CAM model provides the possibility for evaluation of drug effects on vessels in a living organism but does not allow a comprehensive assessment of all drug effects. For these reasons, animal models remain irreplaceable whereby the CAM model should be considered a transition model from in vitro to in vivo experiments facilitating the generation of relevant insights, which are the prerequisite for an efficient planning of in vivo experiments involving animal models. Due to methodological limitations. our preliminary study does not provide answers on the question concerning the stability of the nanodrug within the cerebrospinal fluid space (CSF) and interactions with the CSF clearance, which is the objective of future in vivo experiments in a rat model. In this preliminary study, we were able to provide a proof of the concept for production of a nimodipine-loaded nanodrug with on-demand drug release by ultrasound application, which was the prerequisite to plan further experiments in an animal model. Another objective in future in vivo experiments is the dose determination of the nanodrug for an effective resolution of cerebral vasospasm in a subarachnoid hemorrhage rat model. Furthermore, the definition of the ultrasound parameters for application through the skull is also one of the objectives for future research alongside with experiments for assessment of antivasospastic as well as toxicity-related side effects of the nanodrug after intrathecal application.

## Conclusion

In this experimental study, we were able to provide a proof of the concept for in vivo vasospasm induction in the CAM model with subsequent resolution by ultrasound application in the CAM model with ultrasound-mediated nimodipine release from nanocarriers. Even though the mechanism governing these effects has to be determined yet, the findings of our study are encouraging with respect to further evaluation of this concept in a rat SAH model.
